# Predictors of Falls in Older Adults With and Without Dementia

**DOI:** 10.1093/geroni/igab046.1729

**Published:** 2021-12-17

**Authors:** Safiyyah Okoye, Chanee Fabius, Jennifer Wolff

**Affiliations:** 1 Johns Hopkins University, Baltimore, Maryland, United States; 2 Johns Hopkins Bloomberg School of Public Health, Baltimore, Maryland, United States

## Abstract

Persons living with dementia (PLWD) have up to twice the risk for falling and three-times the risk of serious fall-related injuries as those without dementia. Falls are a leading cause of hospitalizations among PLWD, who are more likely to incur high costs and experience negative health consequences (e.g, delirium, in-hospital falls) than persons without dementia. Few studies have examined risk factors for falls comparing Americans with and without dementia. We used data from the 2015 and 2016 rounds of the National Health and Aging Trends Study (n=5,581) to prospectively identify risk factors for a single fall and recurrent (2+) falls over a 12-month period among community-living older adults ≥65 years with and without dementia in a series of bivariate logistic regressions. Overall, we identified fewer predictors of single or recurrent falls among PLWD compared to persons without dementia. For example, socioeconomic indicators (e.g., income, financial hardship) predicted recurrent falls in persons without dementia, but not in PLWD. Among PLWD, falling in the previous year was associated with both single (odds ratio (OR): 3.38, 95% confidence interval (CI): 1.77, 6.49) and recurrent falls (OR: 6.19, 95% CI: 3.50, 10.93). PLWD who experienced recurrent falls were also more likely to be identified as having a fear of falling (OR: 2.17, 95% CI: 1.33, 3.54), physical function impairments, depression symptoms (OR: 2.23, 95% CI: 1.34, 3.71), and anxiety symptoms (OR: 1.73, 95% CI: 1.14, 2.62). Further study of fall-risk factors could inform screening, caregiver education and support, and prevention strategies for PLWD.

